# PacBio next-generation sequencing uncovers Apicomplexa diversity in different habitats

**DOI:** 10.1038/s41598-023-40895-y

**Published:** 2023-09-12

**Authors:** Mahmoud Gad, Mariam E. Fawzy, Ahmad Z. Al-Herrawy, Sayeda M. Abdo, Noura Nabet, Anyi Hu

**Affiliations:** 1 Environmental Parasitology Laboratory, Water Pollution Research Department, National Research Centre, Giza, 12622 Egypt; 2 Water Pollution Research Department, National Research Centre, Giza, 12622 Egypt; 3 Hydrobiology Laboratory, Water Pollution Research Department, National Research Centre, Giza, 12622 Egypt; 4 Zoology Department, Faculty of Science, Menoufia University, Menofia, Egypt; 5 CAS Key Laboratory of Urban Pollutant Conversion, Institute of Urban Environment, Chinese Academy of Sciences, Xiamen, 361021 China

**Keywords:** Microbiology, Environmental sciences

## Abstract

The phylum Apicomplexa comprises a large group of intracellular protozoan parasites. These microorganisms are known to infect a variety of vertebrate and invertebrate hosts, leading to significant medical and veterinary conditions such as toxoplasmosis, cryptosporidiosis, theileriosis, and eimeriosis. Despite their importance, comprehensive data on their diversity and distribution, especially in riverine environments, remain scant. To bridge this knowledge gap, we utilized next-generation high-throughput 18S rRNA amplicon sequencing powered by PacBio technology to explore the diversity and composition of the Apicomplexa taxa. Principal component analysis (PCA) and principal coordinate analysis (PCoA) indicated the habitat heterogeneity for the physicochemical parameters and the Apicomplexa community. These results were supported by PERMANOVA (*P* < 0.001), ANOSIM (*P* < 0.001), Cluster analysis, and Venn diagram. Dominant genera of Apicomplexa in inlet samples included *Gregarina* (38.54%), *Cryptosporidium* (32.29%), and *Leidyana* (11.90%). In contrast, outlet samples were dominated by *Babesia*, *Cryptosporidium*, and *Theileria*. While surface water samples revealed 16% and 8.33% relative abundance of *Toxoplasma* and *Cryptosporidium*, respectively. To our knowledge, the next-generation high throughput sequencing covered a wide range of parasites in Egypt for the first time, which could be useful for legislation of the standards for drinking water and wastewater reuse.

## Introduction

Rivers and wastewater treatment plants (WWTPs) harbor a diverse microbial community that goes beyond prokaryotes, encompassing eukaryotic entities such as protozoa, fungi, algae, and microscopic metazoans^1–3^. The microbial communities not only affect the efficiency and stability of the WWTP but also may impact human health and the environment upon discharge of wastewater into the receiving environment^4,5^. Among the microbial communities, Apicomplexa stands out as a fundamentally parasitic microeukaryotic group. Apicomplexa is a diverse group of parasitic protists that comprises over 6000 known and potentially many more unknown species. As obligate parasites, they rely on host organisms for survival and can potentially infect a wide range of vertebrates and several invertebrates^6^. This group includes numerous gut-associated taxa, notably *Cryptosporidium* and *Giardia*, known to pose significant health risks to both humans and animals^6–8^. One of the essential roles of wastewater treatment methods is eliminating parasitic species, such as *Cryptosporidium* and *Toxoplasma*^9–11^. Yet, there remains a limited understanding of the efficacy of WWTPs in eliminating apicomplexan species as well as their diversity across different environments.

Many apicomplexans exhibit high host specificity, while some have a more generalist nature. From a medical perspective, they are among the most significant eukaryotic parasites. For instance, *Cryptosporidium* has been recognized as the second most frequently detected enteric pathogen in infants from developing nations^12^. Cryptosporidiosis can also cause an epidemic of diarrhea in developed countries^13^. In a comprehensive study by the Global Enteric Multicentre Study (GEMS) focusing on pediatric diarrheal disease in sub-Saharan Africa, it was found that children under 2 years of age experience approximately 2.9 million *Cryptosporidium* infections each year. Notably, the risk of mortality caused by cryptosporidiosis doubles for children between the ages of 1 and 2 years^12^. *T. gondii*, responsible for toxoplasmosis, has a global footprint, infecting approximately 20% of humans worldwide. Remarkably, *T. gondii* can infect almost all mammalian and avian species^14^.

Various *Eimeria* species, which are specialized to infect particular hosts, can be detected in multiple vertebrates, including poultry and rabbits. Theileriosis, caused by species like *Theileria parva* and *Theileria annulata*, leads to significant economic losses in cattle farming^12,15^. Several *Babesia* species cause babesiosis in various animals including cattle, dogs, horses, and rarely humans^12,15^. The diseases caused by these pathogens (i.e., *Theileria* and *Babesia*) can lead to economic losses as they may cause low milk production, poor growth, and mortality in infected animals. Although developed countries have successfully implemented vector eradication programs, tropical and subtropical countries still suffer from economic losses due to these diseases. Alternative approaches are needed for effective control of piroplasmosis since vector eradication is not feasible in most tropical regions. Commercially available vaccines, containing live attenuated *B. bovis* and *B. bigemina*, have been widely used in the New World and Australia^15^.

In Egypt, earlier researches reported only a limited number of Apicomplexa species in aquatic environments, such as *Cryptosporidium*, *Cyclospora*, and *Isospora*, using microscope, PCR, real-time PCR, and multiplex PCR^16–18^. These methods typically focus on identifying a narrower range of parasites. In contrast, the use of next-generation high-throughput sequencing offers a more comprehensive overview of a wide range of parasites. So, we employed next-generation high throughput 18S rRNA amplicon sequencing based on PacBio technology to reveal the diversity and composition of Apicomplexa and its spatial variation between different habitats (inlet and outlet of WWTP and the Nile River water samples).

## Material and methods

### Samples collections

**Figure 1:**
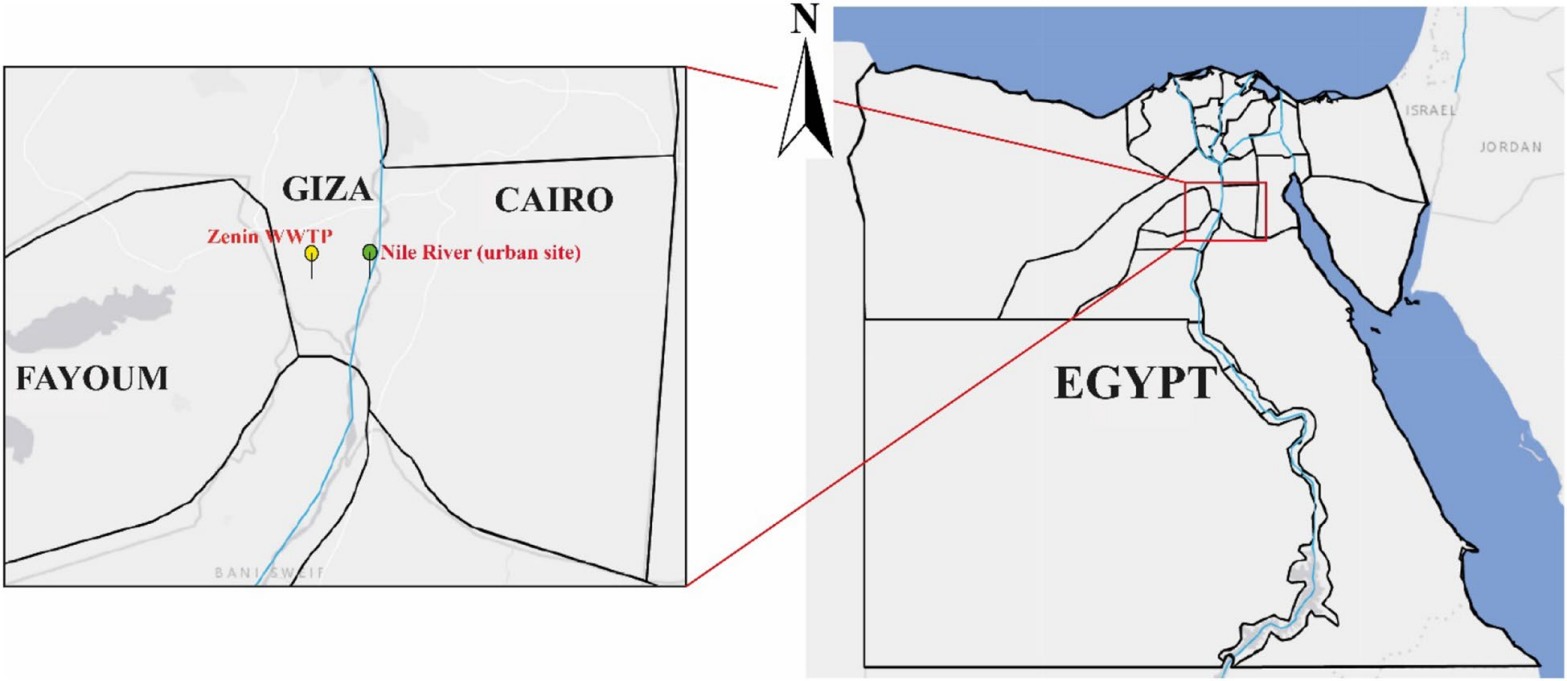
map showing the sampling sites at Nile River and Zenin WWTP.

A total of 34 samples were collected from three habitats: inlet (n = 8) and outlet (n = 8) of Zenin wastewater treatment plant, and an urban location at the Nile River (n = 18). From each habitat, three representative samples were chosen randomly for next-generation high-throughput 18S rRNA PacBio sequencing. To analyze the microeukaryotic community, water samples (~ 1000 mL) were filtered through 0.22 μm Sterivex-GP filters (Millipore, Bedford, MA, USA), and the filters were kept at − 80 °C until DNA extraction. Zenin Wastewater Treatment Plant (WWTP), located in Giza, Egypt (Fig. 1), utilizes activated sludge technology. Established in 1965, it spans 96 acres and was originally designed with a capacity of 330,000 m^3^/day^19^. Presently, Zenin WWTP operates with an actual capacity ranging between 400,000 and 550,000 m^3^/day.

### Physicochemical analysis

Physicochemical parameters were measured according to the International Standard Methods for Water and Wastewater^20^. Briefly, a AD 360 DO meter (Adwa Instruments, Inc, Europe) was used to determine the temperature, dissolved oxygen (DO) and DO saturation of the water samples in situ. pH was measured using a bench pH meter Jenway model 3510. Electric conductivity (EC) and total dissolved solids (TDS) were measured by 4510 Jenway conductivity meter. Chemical oxygen demand (COD) was measured according to dichromate method 5210-D using digestion for two hours at 150 °C on HANNA COD reactor and followed by colorimetric measurement using Lovibond spectrodirect after cooling. Total Kjeldahl Nitrogen (TKN) was measured using mercuric sulfate digestion method followed by titration method 4500-Norg, ammonia–nitrogen (NH_3_–N) was measured titrimetric according to method 4500-NH_3_, nitrite–nitrogen (NO_2_–N) was detected according to colorimetric method 4500-B and nitrate–nitrogen (NO_3_–N) by modified sodium salicylate method according to Scheiner^21^. Total nitrogen (TN) was calculated as the sum of organic nitrogen, NO_2_–N, NO_3_–N, and NH_3_–N. Total phosphorous (TP) was measured according to the method (4500-C).

### DNA extraction, 18S rRNA gene full-length high-throughput sequencing

Environmental DNA was extracted from the collected samples using the DNeasy PowerLyzer PowerSoil Kit (QIAGEN, USA). The DNA concentration was quantified using the Qubit 2.0 fluorometer with the Qubit dsDNA BR assay kit (Life Technologies, Grand Island, NY, USA). The 18S RNA full-length genes were amplified using the primer pairs Euk-A and Euk-B^22^. PCR reactions were conducted in triplicate for each sample, with 25 μL reaction volume containing 5 μL TransStart FastPfu Buffer (5×), 2 μL dNTPs (2.5 mM), 0.5 μL TransStart FastPfu DNA Polymerase (2.5 units/μL, TransGen Biotech, Beijing, China), 0.4 μM of forward and reverse primers, and 10 ng of template DNA. PCR amplification involved an initial denaturation at 95 °C for 5 min, followed by 30 cycles of 95 °C for 30 s, 55 °C for 45 s, and 72 °C for 90 s, and a final extension at 72 °C for 10 min. PCR products were purified using the QIAquick@ Gel Extraction Kit (Qiagen, Santa Clarita, CA, USA), and sequencing libraries were prepared using the SMRTbellTM Template Prep Kit (Pacific Biosciences, Menlo Park, CA, USA) according to the manufacturer's instructions. Finally, the sequencing was conducted on a PacBio Sequel II platform (Creative-proteomics, NY, USA).

### Sequence analysis

The 18S rRNA gene sequences of microeukaryotes were subjected to quality trimming using PacBio SMRT portal v2.3.0. Reads that did not meet the minimum pass of ≥ 3, minimum predicted accuracy of ≥ 90%, and length criteria of 1340–1640 bp were discarded. After quality control, an average of 15,125 high-quality reads per sample remained, which were used for chimera-checking and clustering into operational taxonomic units (OTUs) at the 97% identity cutoff using UPARSE v7.0.1001^23^. The representative sequence of each OTU was classified using the SILVA database v138 and RDP classifier at a confidence threshold of 80%^24^. To standardize the uneven sequencing effort, all samples were randomly subsampled to the smallest library sizes with 14,000 reads for microeukaryotic communities.

### Statistical analysis

Principal Component Analysis (PCA) based on the Euclidean distance was used to characterize the patterns of physicochemical parameters in different habitats. Principal Coordinate Analysis (PCoA) based on the Bray–Curtis distance index was employed for mapping the apicomplexan OTUs in different environments. In order to show the relationship between the apicomplexan community (response group) and physicochemical parameters (explanatory group) in different habitats, distance-based Redundancy Analysis (db-RDA) was employed. The significance of differences in physicochemical parameters among the habitats was tested using permutational multivariate analysis of variance (PERMANOVA) and analysis of similarity (ANOSIM)^25^. The statistical analyses and visualization were performed using PRIMER v.7.0.21 (Quest Research Limited, Auckland, New Zealand) and R v4.1.0 (https://www.r-project.org/).

## Results and discussion

**Figure 2:**
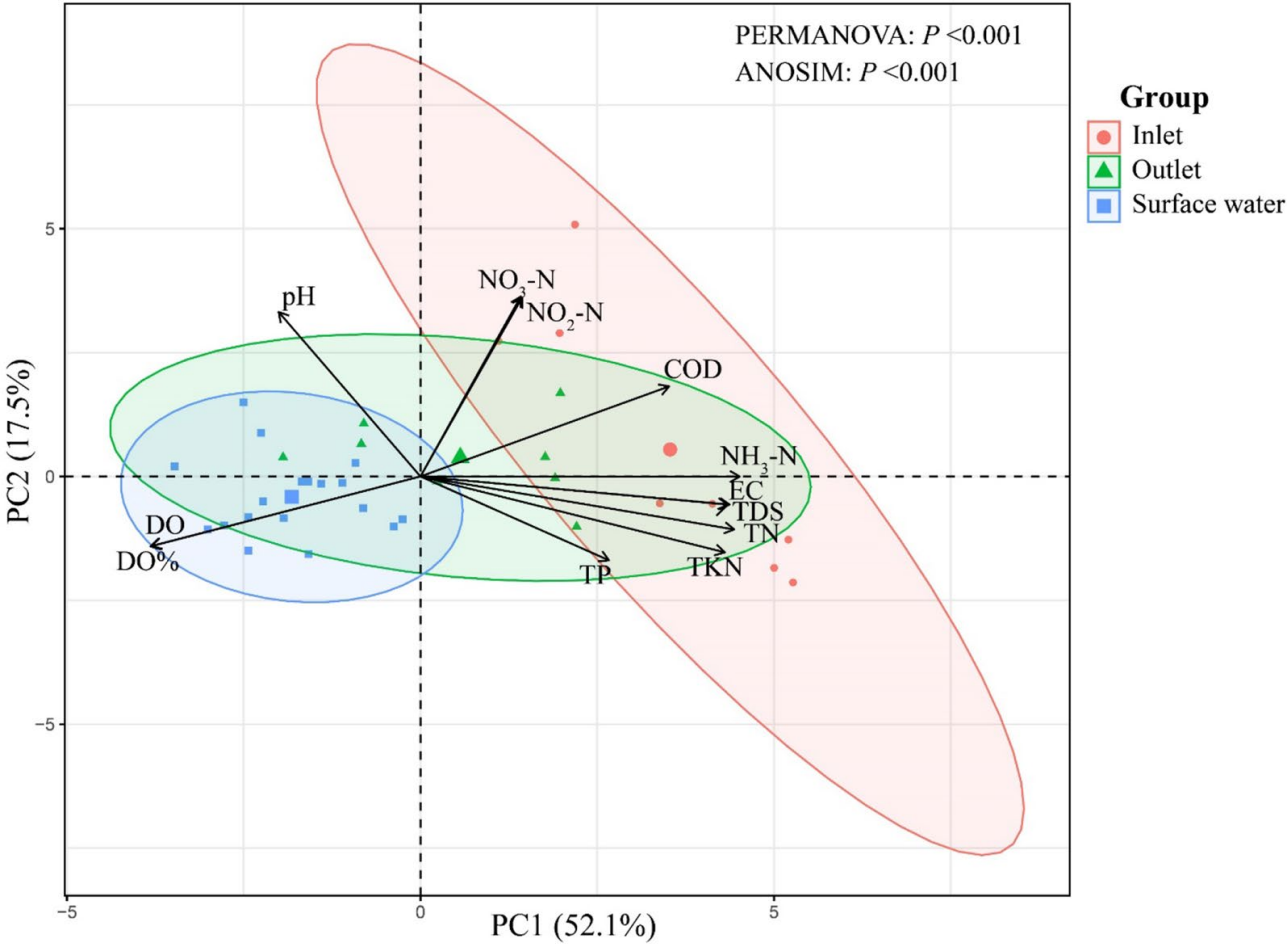
Principal component analysis (PCA) plot for spatial variation of physicochemical parameters between different habitats (principal components (PC1 and PC2) explained 69.6% of the total variation).

The total number of microeukarytic OTUs was 52,214, with 136,127 high-quality reads. PCA results indicated clustering of physicochemical parameters based on habitat differences, especially between inlet and surface water samples. Interestingly, some lower-quality outlet samples were similar to inlet samples, while higher-quality samples resembled surface water samples (Fig. 2). Significant differences in environmental parameters during the wastewater treatment process and in the receiving environment were confirmed by PERMANOVA (*P* < 0.001) and ANOSIM (*P* < 0.001). Most notably, the difference in physicochemical parameters between inlet and surface water was more pronounced (ANOSIM; R = 0.94) than differences between inlet and outlet or between outlet and surface water (ANOSIM; R < 0.67) (Table 1).

**Table 1:** PERMANOVA and ANOSIM to test the significance of the differences in physicochemical parameters between the habitats.

Group	PERMANOVA		ANOSIM	
	t	*P*	R	*P*
Inlet vs. outlet	2.10	0.004	0.38	0.003
Inlet vs. surface water	5.19	< 0.001	0.94	< 0.001
Outlet vs. surface water	3.27	< 0.001	0.67	< 0.001

During the study period, the physicochemical parameters of the inlet wastewater samples obtained from Zenin WWTP were marked by the presence of organic constituents, demonstrated by COD, and the presence of abundant nutrients such as TKN, NH_3_–N, NO_2_–N, NO_3_–N, TP, and TN. Their corresponding average concentrations were 270 mgO_2_/L, 34.97 mg/L, 18.5 mg/L, 0.22 mg/L, 1.8 mg/L, 2.59 mg/L and 36.99 mg/L, respectively. The results of treated effluent indicated the average percentage of removal was more than 82% of COD, 74% of TP, and almost 60% of TN content. Biodegradation of organic matter in the activated sludge process takes place by the action of bacteria, protozoa, and metazoan^26^. Bacteria dominated all other biota groups in the activated sludge process, and influenced the process of mineralization and degradation of organic and inorganic nutrients^26,27^. The microeukaroytes, such as free-living protozoa, feed on dispersed bacteria and suspended particles to purify and clean the wastewater^26^.

**Figure 3:**
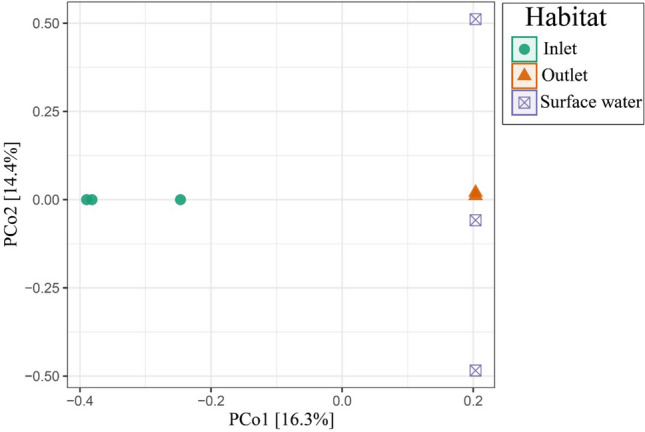
Principal coordinate analysis (PCoA) based on Bray Curtis similarity index showing the β-diversity patterns of the Apicomplexa community.

**Figure 4:**
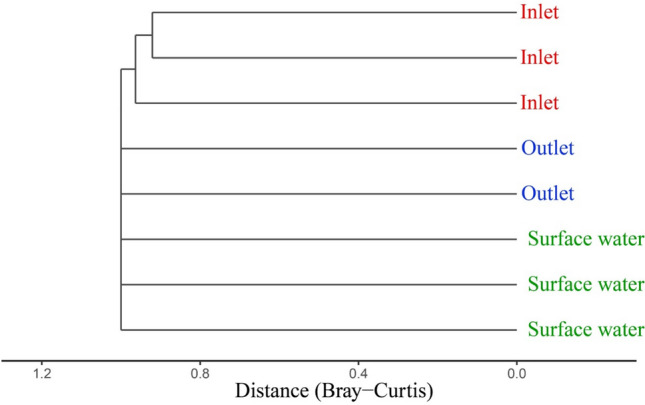
Cluster analysis of Apicomplexa OTUs in different environments.

**Figure 5:**
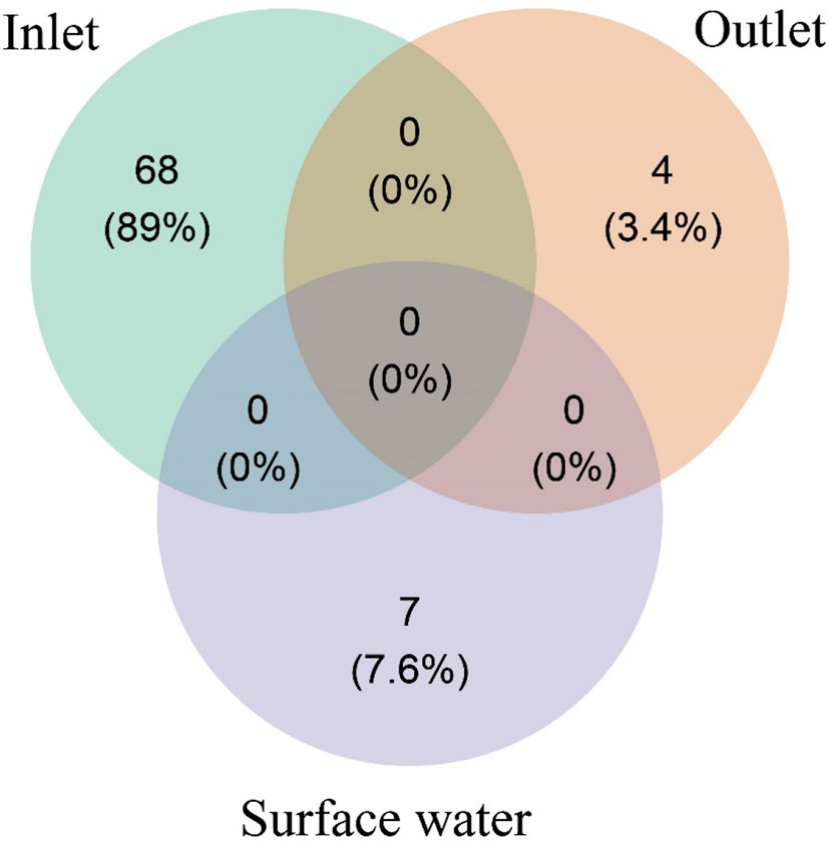
Venn shape shows the unique and shared Apicomplexa OTUs in different habitats.

Overall, the PCoA revealed that Apicomplexa community exhibited a significant difference between different habitats. The inlet samples were clustered far from the outlet and surface water samples (Fig. 3). The results of Cluster Analysis (Fig. 4) supported those of PCoA (Fig. 3). The apicomplexan species represented a lower proportion (< 0.01%) in the microeukaryotic communities. Comparable results regarding microeukaryotic communities including the parasitic Apicomplexa were noted in various habitats both in China^25,28^ and in USA^29^. The Venn diagram indicated no shared apicomplexan species across habitats. While 68 (89%) unique apicomplexan species were observed in the inlet samples, lower unique species were observed in the outlet (n = 4) and surface water samples (n = 7) (Fig. 5). The study's findings suggest that the physicochemical parameters of various habitats impacted the apicomplexan community. Further studies are needed to investigate the specific factors that drive the distribution of apicomplexan species in different aquatic environments.

**Figure 6:**
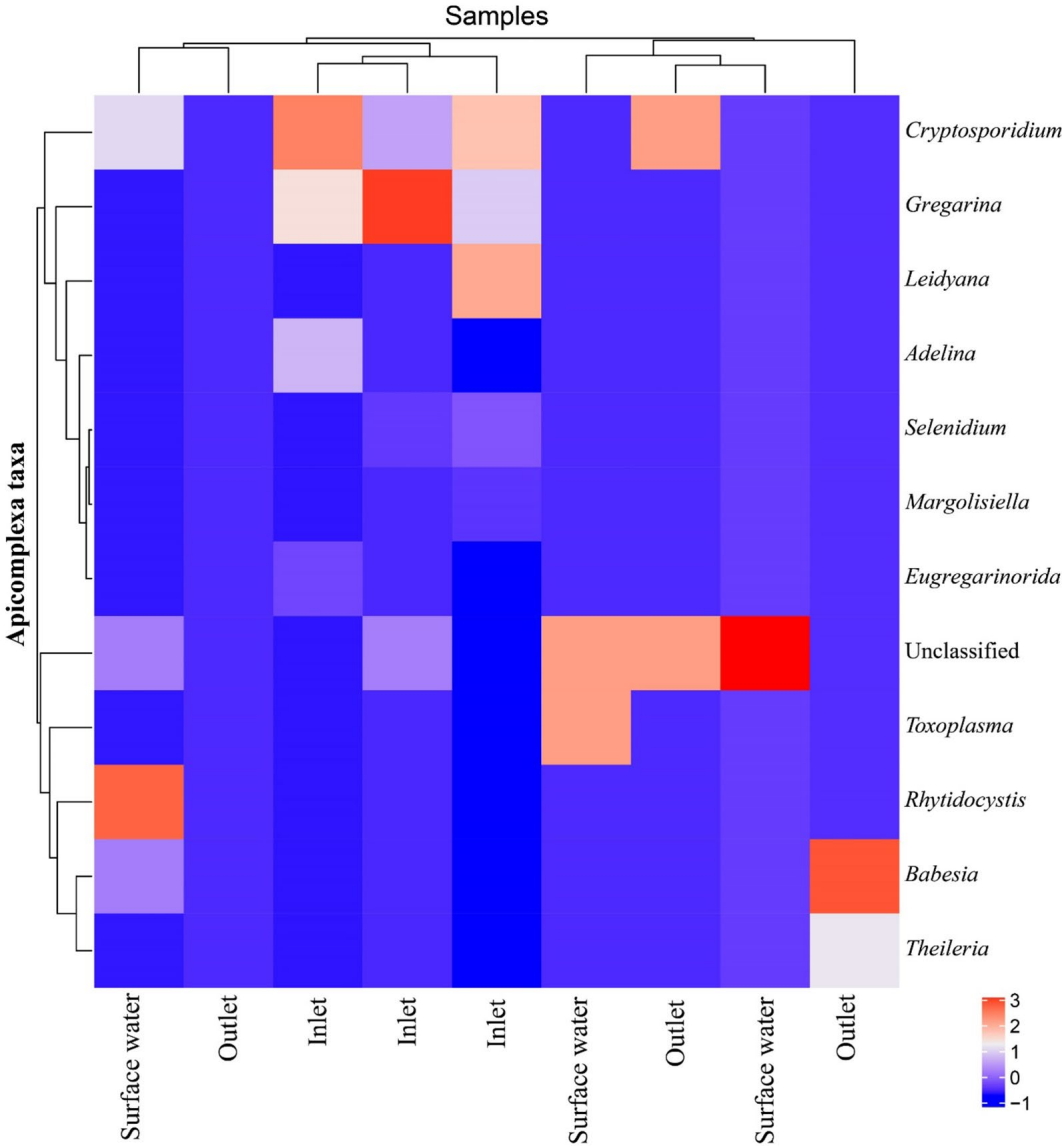
Heatmap showing the distribution of Apicomplexa taxa in different environments.

The heatmap showed that the inlet samples comprised more apicomplexan genera than the outlet and surface water samples. The significant decrease in parasite abundance from high levels in the raw sewage (inlet) to low levels in the outlet and the receiving surface water environment (Nile River water) indicated the successful removal of parasites during the wastewater treatment process (Fig. 6). The results are consistent with a previous study by Freudenthal et al.^30^, which highlighted the importance of wastewater treatment technology in reducing potential health risks associated with treated wastewater reuse. Effective removal and inactivation of pathogenic microorganisms are essential in ensuring the safety of treated wastewater reuse. Earlier studies have identified ciliates (protists) and rotifers (metazoans) as the most likely predators for small protists and bacteria in WWTPs^31,32^. Additionally, the network analysis results revealed associations between parasitic protists (e.g. *Dientamoeba*, *Entamoeba*, and *Giardia*) and their potential predators (ciliates and rotifers), which might be attributed to predation^30^.

**Figure 7:**
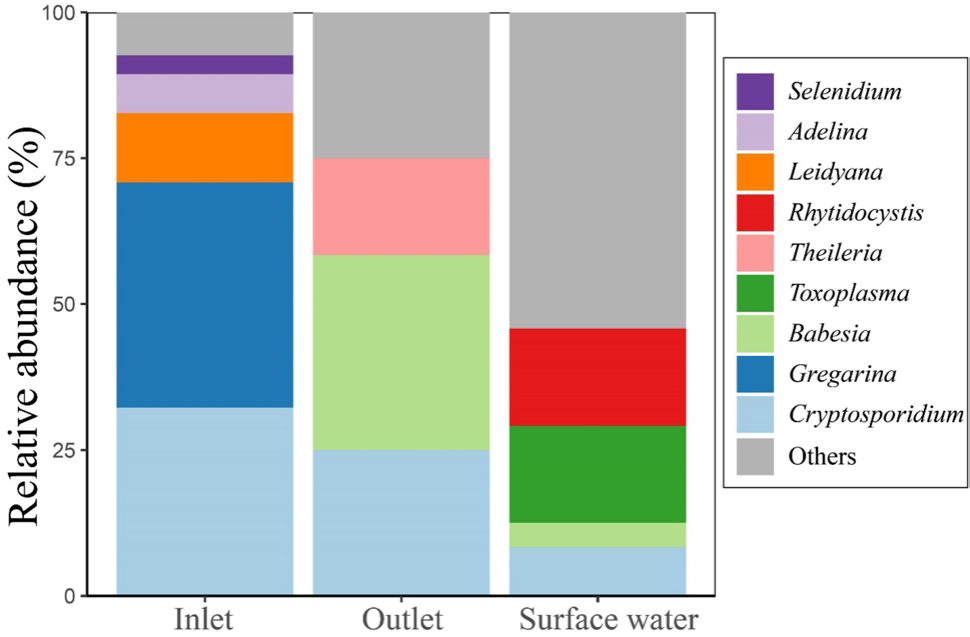
Taxonomic composition of the Apicomplexa community. Others refer to unclassified and less contributed taxa.

The taxonomic composition of the apicomplexan community revealed that the most dominant parasites in the inlet samples were *Gregarina* (relative abundance; 38.54%)*, Cryptosporidium* (32.29%)*,* and *Leidyana* (11.90%)*. Babesia, Cryptosporidium*, and *Theileria* were the dominant taxonomic groups of Apicomplexa communities in the outlet samples, accounting for 33.33%, 25%, and 16.67%, respectively (Fig. 7). Apicomplexa (~ 57.2% in inlet and ~ 46.9% in outlet) were the most dominant protozoa in the New Zealand’s wastewater, followed by Cercozoa, Evosea, Euglenozoa, Microsporidia, and Fornicata^5^. At the genus level, Apicomplexa in wastewater was mainly represented by *Plasmodium*, *Toxoplasma*, *Cryptosporidium*, *Giardia*, *Babesia*, and *Theileria*^5^. Similar to the study of Ariyadasa et al.^5^, the relative abundance of *Cryptosporidium* decreased in outlet compared to the inlet. In earlier researches, conventional detection methods consistently revealed that wastewater was a potential hotspot for parasites^33–35^.

The current study highlights the diversity and abundance of the parasitic apicomplexans, particularly in the Nile River using next-generation sequencing technology. In surface water samples, the harmful parasite (i.e., *Toxoplasma*) appeared with relative abundance around 16%, and lower relative abundance of *Cryptosporidium* (8.33%) was observed comparing to the other environments (Fig. 7). Loads of enteric parasites of Apicomplexa in wastewater may be due to the population density, per capita water consumption, survival of the excreted stage (e.g., cysts or oocysts) in wastewater^36^, habits, and presence of cats or other domestic animals in the area^36,37^. Individuals infected with *Cryptosporidium* shed large numbers of oocysts in feces (up to 10^9^ oocysts per stool) that persist in cold and moist environments for up to 6 months^38,39^. In 2019, Maritz et al. identified a variety of parasitic protist taxa, including *Entamoeba*, *Blastocystis*, and *Trichomonas* in sewage samples using 18S rRNA amplicon sequencing^40^. While a wide parasitic protists range, including *Blastocystis*, *Entamoeba, Trichomonas, Dientamoeba*, *Guttulinopsis*, *Giardia*, and *Rosculus* were identified in WWTPs using shotgun metagenome^30^*.* Gad et al. used machine learning approaches, such as SourceTracker and Fast Expectation–mAximization Microbial Source Tracking (FEAST) to track Apicomplexa OTUs in the Changle River watershed. Their research revealed that a considerable portion of exogenous Apicomplexa from both the main channel and tributaries may have originated from untreated domestic and swine wastewater, as well as treated wastewater (i.e., outlets of WWTPs)^28^. In line with our findings, *Cryptosporidium* species were also detected in the Changle River (China), particularly in the river’s tributary^28^. These parasitic microeukaryotes can persist in surface waters for extended periods, even up to several months^41^. Therefore, additional studies are needed to assess the potential public health hazards associated with the presence of parasitic Apicomplexa, including *Cryptosporidium* and *Toxoplasma*, in the Nile River. Moreover, the monitoring of community composition dynamics of these parasites may be an important new tool to assist in managing and optimizing the removal of contaminants from wastewater and to inform future risk assessments for improving human, animal, and environmental health.

## Conclusion

Utilizing next-generation high throughput 18S rRNA amplicon Pacbio sequencing to investigate Apicomplexa in various aquatic habitats in Egypt. This novel approach enabled us to detect a wide range of apicomplexan parasites. Our findings revealed the presence of some apicomplexan species, such as *Cryptosporidium*, in the outlet (treated sewage samples) and surface water samples from the Nile River, which may indicate insufficient wastewater treatment and pose health threats to humans and animals. Our study has implications for the development of legislation concerning drinking water and wastewater reuse standards at the national level based on WHO recommendations. Further studies are required to explore the diversity and composition of microeukaryotic parasites in other habitats such as drinking water and swimming pools using amplicon sequencing and shotgun metagenomics technologies.

## Data Availability

The raw sequence data of 18S rRNA genes was deposited in the NCBI short reads archive database under BioProject number PRJNA952662.
